# Studying the Impact of the Temperature and Sorbed Water during Microwave-Induced *In Situ* Amorphization: A Case Study of Celecoxib and Polyvinylpyrrolidone

**DOI:** 10.3390/pharmaceutics13060886

**Published:** 2021-06-15

**Authors:** Nele-Johanna Hempel, Matthias M. Knopp, Korbinian Löbmann, Ragna Berthelsen

**Affiliations:** 1 Department of Pharmacy, University of Copenhagen, 2100 Copenhagen, Denmark; nele.hempel@sund.ku.dk (N.-J.H.); korbinian.loebmann@sund.ku.dk (K.L.); 2 Bioneer: FARMA, Department of Pharmacy, University of Copenhagen, 2100 Copenhagen, Denmark; mmk@bioneer.dk

**Keywords:** *in situ* amorphization, microwave radiation, amorphous solid dispersion, dissolution, celecoxib, polyvinylpyrrolidone

## Abstract

Microwave-induced *in situ* amorphization of a drug into a polymeric amorphous solid dispersion (ASD) has been suggested to follow a dissolution process of the drug into the polymeric network, at temperatures above the glass transition temperature (*T_g_*) of the polymer. Thus, increasing the compact temperature, above the *T_g_* of the polymer, is expected to increase the rate of drug dissolution in the mobile polymer, i.e., the rate of amorphization, in a direct proportional fashion. To test this hypothesis, the present study aimed at establishing a linear correlation between the compact temperature and the rate of drug amorphization using celecoxib (CCX) and the polymers polyvinylpyrrolidone (PVP) 12 and PVP17 as the model systems. Water sorbed into the drug–polymer compacts during 2 weeks of storage at 75% relative humidity was used as the dielectric heating source for the present drug amorphization process, and therefore directly affected the compact temperature during exposure to microwave radiation; the loss of water during heating was also studied. For this, compacts prepared with 30 wt% CCX, 69.5 wt% PVP12 or PVP17 and 0.5 wt% magnesium stearate (lubricant) were conditioned to have a final water content of approx. 20 wt%. The conditioned compacts were exposed to microwave radiation for 10 min at variable power outputs to achieve different compact temperatures. For compacts containing CCX in both PVP12 and PVP17, a linear correlation was established between the measured compact end temperature and the rate of drug amorphization during 10 min of exposure to microwave radiation. For compacts containing CCX in PVP12, a fully amorphous ASD was obtained after 10 min of exposure to microwave radiation with a measured compact end temperature of 71 °C. For compacts containing CCX in PVP17, it was not possible to obtain a fully amorphous ASD. The reason for this is most likely that a fast evaporation of the sorbed water increased the *T_g_* of the conditioned drug–polymer compacts to temperatures above the highest reachable compact temperature during exposure to microwave radiation in the utilized experimental setup. Supporting this conclusion, evaporation of the sorbed water was observed to be faster for compacts containing PVP17 compared to compacts containing PVP12.

## 1. Introduction

*In situ* amorphization is a novel approach to increase the oral bioavailability of poorly water-soluble drugs [1,2,3]. *In situ* amorphization describes the process of transforming a crystalline state of a drug into an amorphous form by forming an amorphous solid dispersion (ASD) within the final dosage form (e.g., a tablet/compact) either in the final manufacturing step or immediately prior to administration [2,4,5,6]. Utilizing the concept of *in situ* amorphization, a physical mixture of a crystalline drug and a polymer can be manufactured into a final dosage form by using cheap, fast and standard direct tableting protocols. It is advantageous to use a crystalline form of the drug, compared to an amorphous form, in terms of physical stability and manufacturability. For example, the poor flowability of an amorphous powder is not of concern for a crystalline powder [7]. After manufacturing, the crystalline drug can be transformed into the amorphous form, allowing the final dosage form to have the characteristic advantages of the amorphous drug such as, e.g., higher solubility and faster dissolution rate compared to the crystalline form [8,9].

*In situ* amorphization has been demonstrated by immersion in water, heat convection, microwave radiation and laser radiation [2,4,5,6,10,11,12,13]. Presently, microwave or laser radiation appear to be the most promising approaches to achieve *in situ* amorphization, as these, so far, are the only approaches for which complete amorphization has been reported [2,13]. Microwave-induced *in situ* amorphization relies on the absorption of microwave radiation by dielectric excipients inside the final dosage form. The absorption of microwave radiation by a substance is specified by the dielectric properties of the substance. A common dielectric molecule is water, as the dipoles of water can easily adapt to the alternating electromagnetic field at 2.45 GHz, which is the fixed frequency most household microwave ovens work at [14,15,16,17]. The movement of the dielectric molecules, aligning to the electromagnetic field, causes friction which results in the generation of heat. Usually, the absorption of microwave radiation by drugs and polymers inside compacts can be neglected with respect to heat generation, as their dielectric properties are poor [16,17]. Examples of dielectric molecules that have been used for *in situ* amorphization are water (including inorganic crystal hydrate salts), glycerol and polyethylene glycol [5,6,12,16,17,18,19].

Several studies in the field of microwave-induced *in situ* amorphization have shown that water, sorbed into compacts, acts both as a plasticizer (i.e., a mobility enhancer), and an enabling dielectric excipient (i.e., a dielectric heating source) [1,2,4,20,21]. The plasticizer function is vital as the rate of amorphization has been shown to increase significantly at temperatures above the glass transition temperature (*T_g_*) of the pure polymer and the forming ASD, when the mobility of polymer increases and its viscosity decreases [6,21].

In previous studies, several factors have been shown to influence the rate of the microwave-induced *in situ* amorphization. For example, it was shown that reducing the particle size of the drug and the polymer (i.e., increasing the surface area of both the drug and polymer particles), and increasing the temperature above the *T_g_* of the polymer, increased the rate of amorphization [2]. Furthermore, it has been shown that decreasing the viscosity of the polymer, e.g., by using a lower molecular weight polymer or higher temperatures obtained during exposure, increases the rate of *in situ* amorphization [6]. All this data supports the hypothesis that microwave-induced *in situ* amorphization can be described as a dissolution process of the drug into the mobile polymer at temperatures above the *T_g_* of the polymer. Accordingly, the rate of amorphization may theoretically be described by the Noyes–Whitney equation (Equation (1)) [22].

(1) $$Noyes-Whitney\;Equation:\;\frac{dm}{dt}=A*\frac{D}{h}*\left(C_{s}-C_{b}\right);$$

(2) $$Diffusion\;coefficient:\;D=\frac{kT}{6\pi \eta r}$$

*dm/dt* = solute dissolution rate;
*m* = mass of dissolved material;
*t* = time;
*A* = surface area of the solute particle;
*h* = thickness of the diffusion layer;
*C_s_* = particle saturation concentration;
*C_b_* = concentration in the bulk solution;
*D* = Diffusion coefficient of the solute in solution;
*k* = Boltzmann constant;
*T* = absolute temperature;
*η* = viscosity of the solvent;
*r* = radius of the solute molecule.

From the Noyes–Whitney equation, it can be seen that the rate of dissolution (i.e., the rate of *in situ* amorphization of the drug into the mobile polymer) is linearly dependent on the diffusion coefficient, which according to the Stokes–Einstein equation (Equation (2)) is linearly correlated with temperature.

Based on the hypothesis that the rate of *in situ* amorphization can be described by the Noyes–Whitney equation (Equation (1)), the first aim of the present study was to study the relation between the compact temperature (at temperatures above the *T_g_* of the polymer) and the rate of *in situ* amorphization upon exposure of compacts to microwave radiation. For this, compacts containing 30 wt% CCX, 69.5 wt% PVP12 or PVP17 and 0.5 wt% magnesium stearate (lubricant) were exposed to microwave radiation after a conditioning period of 2 weeks at 75% relative humidity and room temperature. A drug load of 30 wt% CCX was chosen for the study, as a previous study had shown complete amorphization of compacts containing 30 wt% CCX and PVP12 after exposure to microwave radiation for 10 min using a household microwave oven [2]. Additionally, the drug load of 30 wt% CCX in PVP (12 and 17) is below the solubility of CCX in PVP at room temperature, which means that the formation of a fully amorphous and physically stable ASD (upon cooling) should be possible [23].

The role of water evaporation from the conditioned compacts during exposure to microwave radiation was also studied. As previously mentioned, water acts not only as a plasticizer, lowering the *T_g_* of the components in the compacts, but also as a dielectric heating source. However, water will evaporate during exposure to microwave radiation with increasing compact temperature. Depending on the degree of interaction between the sorbed water and the polymers (PVP12 and PVP17), the compact temperature profile, as well as the evaporation of the water, is expected to vary. Free water is not restricted in its mobility, which results in fast heating rates upon exposure to microwave radiation. Sorbed water of a polymer, here PVP, is bound and therefore restricted in its mobility [16,24,25]. As tightly bound water is more mobility restricted compared to loosely bound water, exposing a polymer with tightly bound water to microwave radiation is expected to result in a lower heating rate, but also a lower rate of water evaporation, which means that the *T_g_* of this polymer will increase at a slower rate, as compared to a polymer with loosely bound water.

The degree of interaction between the sorbed water and the polymers PVP12 and PVP17 was determined by the Gordon–Taylor equation (Equation (3)). According to the Gordon–Taylor equation, a difference between the measured and the theoretical *T_g_* indicates an interaction between the two components of the system [26].

(3) $$\mathrm{Gordon}\mathrm{Taylor}\mathrm{Equation}\;T_{g}=\frac{w_{1}*T_{g}1+K*w_{2}*T_{g}2}{w_{1}+K*w_{2}};where\;K=\frac{T_{g}1*p_{1}}{T_{g}2*p_{2}};$$

*T_g_*1 = *T_g_* of PVP12 or PVP17 in K (see Table 1, water-free *T_g_*)
*T_g_*2 = *T_g_* of water = 135 K
*p*_1_ = amorphous density of PVP = 1.18 g/cm^3^ [27,28]
*p*_2_ = density of water = 0.999 g/cm^3^
*w*_1_ = weight fraction of PVP
*w*_2_ = weight fraction of water

Based on the interaction between the sorbed water and PVP (12 or 17), the second aim of the study was to investigate the role of the compact water with respect to *in situ* amorphization and the interplay between polymer plasticization, dielectric heating source and evaporation. For this, the evaporation of sorbed water from the polymers PVP12 and PVP17 was determined by thermogravimetric analysis, and the results were related to the observed *in situ* amorphization of CCX in PVP12 and PVP17.

## 2. Materials and Methods

### 2.1. Materials

Celecoxib (CCX, *M*_w_ = 381.37 g/mol) and magnesium stearate were purchased from Fagron Nordic A/S (Copenhagen, Denmark). Kollidon^®^ 12PF (PVP12, *M*_w_ = 2000–3000 g/mol) and Kollidon^®^ 17PF (PVP 17, *M*_w_ = 7000–11,000 g/mol) were kindly supplied by BASF (Ludwigshafen, Germany). Sodium chloride (NaCl, *M*_w_ = 58.44 g/mol) was purchased from Sigma-Aldrich (St. Louis, MO, USA). All chemicals were used as received.

### 2.2. Compact Preparation and Storage

CCX, PVP12 and PVP17 were sieved using a 125 µm sieve. Only the particle fraction with a diameter <125 µm was used, as a previous study showed that a small particle size of drug and polymer is advantageous for fast microwave-induced *in situ* amorphization [2]. Subsequently, physical mixtures containing 30 wt% CCX, 69.5 wt% PVP12 or PVP17 and 0.5 wt% magnesium stearate were prepared using mortar and pestle. Using 100 ± 2 mg of the physical mixtures, flat-faced cylindrical compacts (Ø 6 mm) were prepared using an instrumented single punch tablet press GTP-1 from Gamlen Instruments (Nottingham, UK) fitted with a 500 kg load cell (CT6–500-022) at a compaction pressure of 35 MPa. After preparation, the compacts were conditioned for 2 weeks at 75% relative humidity (over a saturated NaCl solution) at room temperature. The compacts were weighted before and after the conditioning period to assess the weight gain due to the sorbed water.

### 2.3. In Situ Drug Amorphization Using a Microwave Oven

In order to induce *in situ* amorphization, a Synthos 3000 microwave oven from Anton Paar GmbH (Graz, Austria) was used. The Synthos 3000 has two magnetrons, which radiate unpulsed microwaves at a frequency of 2.45 GHz. The microwave oven is equipped with a 64 MG5 rotor, which allows 3 rpm and the holding of up to 64 samples. Additionally, the microwave oven has a built-in infrared probe for *in situ* temperature measurements. The temperature of each compact was approx. measured every 20th second. As the temperature was constantly monitored, it was adjusted by a feedback mechanism regulating the power output, i.e., the temperature monitoring allowed the setting of a maximum temperature of the microwave oven, which was kept constant. The maximal power output was set to 1000 W. The microwave oven adjusted the power output from 0–1000 W to reach the compact temperature set by measuring the compact temperature as described above. Three compacts were placed in individual sealed glass vials in temperature-recording positions. Empty glass vials were placed in the remaining 13 temperature-recording positions. Additionally, 16 sealed glass vials, each containing 1.5 mL of demineralized water, were placed in non-temperature recording positions to absorb residual microwave radiation. The water-containing vials had an offset to the glass vials containing compacts to avoid passive heating by conduction. Compacts containing PVP12 were continuously exposed to microwave radiation for 10 min to reach compact temperatures of 60, 70, 80 and 82 °C. Compacts containing PVP17 were continuously exposed to microwave radiation for 10 min to reach temperatures of 60, 70 and 80 °C. Each experiment was conducted with triplicate measurements for each compact composition—temperature combination simultaneously (*n* = 3), using the same batch of compacts. The recorded temperature data were interpolated and the mean and standard deviations were calculated. The temperature data were corrected by a technical factor of 1.214 according to the manufacturer; i.e., the technical factor corrects for the difference between the compact temperature and the temperature of the glass vial (bottom), whereas the temperature of the glass vial is measured by the in-built infrared thermometer. The set compact temperatures of 60, 70, 80 and 82 °C (already corrected by the technical factor) responded to average measured compact end temperatures of 51, 59, 70 and 71 °C, respectively.

### 2.4. Quantification of the Degree of Amorphization

The degree of amorphization, before and after exposure to microwave radiation, was quantified using transmission Raman spectroscopy. For this, a Kaiser RXN1 Microprobe from Kaiser Optical Systems (Ann Arbor, MI, USA) was equipped with a PhaT-probe (Pharmaceutical Area Testing) and used in a transmission Raman configuration setup, as described previously by Edinger et al. (2018) [20]. In short, the compacts were placed on a Thorlabs AD127NT adaptor (Newton, NJ, US), which was situated on the excitation fiber. Using a 5× objective connected to the PhaT-probe, the inelastically scattered lights were collected at a distance of 20 mm to the adaptor. Each spectrum had a total acquisition time of 20 s, which equals an average of 5 measurements with an exposure time of 4 s each. The dark frames were subtracted for each measurement. At the excitation fiber output, the wavelength was 785 nm with an excitation power of 200 mW. At a resolution of 5 cm^−1^, the Raman shift was measured from 150 to 1900 cm^−1^. The analysis was conducted using a calibration space and a partial least-squares regression (PLS) model, which was kindly supplied by Edinger et al. (2018) [20]. Distinguishing features present in the spectral region from 705 to 845 cm^−1^ were used to differentiate between the crystalline and the amorphous form of CCX. The calibration space for the PLS model was obtained from 17 different mixtures containing crystalline CCX, amorphous CCX and PVP17. Preprocessing of the data was performed by Savitzky–Golay smoothing and standard normal variate transformation; all data processing was performed in MatLab from MathWorks (Natick, MA, USA) using the PLS toolbox 8.1.1 from Eigenvector Research Inc. (Manson, WA, USA).

### 2.5. Thermal Analysis

Thermal analysis was conducted using a Discovery differential scanning calorimeter (DSC) from TA Instruments (New Castle, DE, USA) at a nitrogen gas purge of 50 mL/min. Compacts exposed to microwave radiation at the highest temperature were ground using mortar and pestle. A total of 2–6 mg of powder was weighed into Tzero aluminum pans, which were closed with hermetically sealing lids.

Determination of the *T_g_* values was performed using modulated DSC (mDSC) at a heating rate of 3 °C/min with an amplitude of 1 °C/50 s. The *T_g_* values of the formed ASD and the polymers containing bulk water were determined by heating from −20 °C to 120 °C. The *T_g_* values of the pure conditioned polymers were determined from −60 °C to 50 °C for PVP12, and −60 °C to 70 °C for PVP17. The *T_g_* values of the water-free polymers were determined by applying a heat–cool–heat cycle. For the water to evaporate during the first heating, the lid was perforated. The polymers were initially equilibrated for 10 min at 120 °C and subsequently equilibrated to −20 °C. Afterwards, a heating to 170 °C was applied. The *T_g_* was defined as the midpoint over the change in heat capacity using the TRIOS software (version 5.1.1) from TA Instruments (New Castle, DE, USA). All *T*_g_ values were determined in duplicates (*n* = 2).

The water content of the pure polymers (*n* = 3) and the water loss of the conditioned polymers (*n* = 1) were determined using a Discovery thermogravimetric analyzer 1 (TGA) from TA Instruments Inc. (New Castle, DE, USA) using a nitrogen gas purge of 25 mL/min. Approx. 10 mg of the pure polymers were placed in an open platinum pan and analyzed at a heating rate of 10 °C/min up to 150 °C.

Using the TGA, the conditioned polymers were also placed in an open platinum pan (approx. 50 mg) and exposed to the heating rate obtained for compacts containing PVP12 or PVP17 that were exposed to microwave radiation at a measured compact end temperature of 70 °C, to study the water evaporation process. Conditioned PVP12 was analyzed at a heating rate of 30.8 °C/min to 60 °C, followed by a heating rate of 1.2 °C/min to 70 °C. Conditioned PVP17 was analyzed at a heating rate of 38.8 °C/min to 60 °C, followed by a heating rate of 1.1 °C/min to 70 °C. The weight loss was determined using the TRIOS software (version 5.1.1).

### 2.6. Solid State Characterization

The solid state characteristics of the compacts, before and after exposure to microwave radiation, were confirmed qualitatively using X-Ray powder diffraction (XRPD). For this, an X’Pert Pro diffractometer from PANalytical (Eindhoven, the Netherlands) using Cu Kα radiation (λ = 1.54187 Å) was used. The diffractograms were recorded at 45 kV and 40 mA from 5 to 30° 2θ, and were analyzed using the X’Pert Data viewer software (version 1.2) from PANalytical (Eindhoven, the Netherlands). Each sample was analyzed once (*n* = 1).

## 3. Results and Discussion

As previously mentioned, it has been suggested that microwave-induced *in situ* amorphization can be described as a dissolution process, which means that the rate of amorphization can be described by the Noyes–Whitney equation (Equation (1)). To further support this hypothesis, the present study aimed to investigate the relationship between the compact temperature and rate of drug amorphization. Furthermore, the role of the evaporation of the sorbed water inside the compact was studied, as the dehydration affects the *T_g_* of the polymer PVP and the forming ASD as well as the dielectric heating.

### 3.1. Characterization of the Compacts after Conditioning

During the conditioning period of 2 weeks at 75% relative humidity, the compacts gained weight due to water sorption of the hygroscopic polymer PVP. The weight gain was determined to be 15.3 ± 0.5 wt% (*n* = 15) for compacts containing PVP12 and 12.8 ± 1.1 wt% (*n* = 12) for compacts containing PVP17. Including the water content of the bulk PVP, 4.1 ± 0.0 wt% for PVP12 and 7.7 ± 0.1 wt% for PVP17 (*n* = 3), the total water content of all the compacts after storage was approx. 20 wt% (19.4 ± 0.5 wt% for PVP12, 20.5 ± 1.2 wt% for PVP17).

As the polymers inside the compacts had a *T_g_* below room temperature after conditioning (Table 1), minor amorphization occurred during the conditioning period, which is in line with findings reported in previous publications [2,4,20]. In the present study, the initial amorphization was found to be 5.0 ± 0.4% (*n* = 3) for compacts containing PVP12 and 0.2 ± 0.1% (*n* = 3) for compacts containing PVP17. The observed difference in the initial degree of amorphization for compacts containing PVP12 and PVP17 is suggested to be due to the fact that PVP12 has a lower *T_g_*, due to lower molecular weight and a lower viscosity at room temperature, as compared to PVP17. Upon conditioning, all the compacts significantly softened due to the high amount of sorbed water.

In order to determine the degree of interaction between the sorbed water and the polymer, the theoretical *T_g_* values were calculated using the Gordon–Taylor equation (Equation (3)) [29]. The measured and the theoretical *T_g_* values of the polymers PVP12 and PVP17, prior to-, and after conditioning, can be found in Table 1.

As can be seen in Table 1, it is apparent that the sorbed water, acting as a plasticizer, significantly lowered the *T_g_* of the two polymers. For both PVP12 and PVP17, the *T_g_* was approx. lowered by 65 °C with the sorption of water in the bulk PVP. For PVP12, it can be seen that a *T_g_* below 0 °C was obtained after conditioning.

When comparing the experimental *T_g_* values with the theoretical *T_g_* values obtained using the Gordon–Tayler equation, it is apparent that the experimentally determined *T_g_* values are lower than the theoretical values, in all cases except for PVP17 after conditioning (Table 1). As a negative deviation from the Gordon–Tayler equation is commonly interpreted as an increase in the mobility of the system, i.e., in this case as the water of the bulk polymer having a highly plasticizing effect on PVP12 and PVP17, the degree of plasticization originating from the water of the bulk PVP was found to be higher for PVP12 compared to PVP17. The different degree of (negative) deviation of the experimentally determined *T_g_* values from the theoretical *T_g_* values suggests differences in the degree of interaction of the water molecules with PVP12 and PVP17. Based on these results (Table 1), it is hypothesized that the ratio between tightly bound and loosely bound water is higher for PVP12 compared to PVP17 [16,25].

For both polymers, the negative deviation between the measured and the theoretical *T_g_* value became smaller after the conditioning period, suggesting a higher degree of loosely bound water in both conditioned polymers as compared to the bulk polymers (Table 1).

### 3.2. Exposure to Microwave Radiation of Compacts Containing PVP12

**Figure 1:**
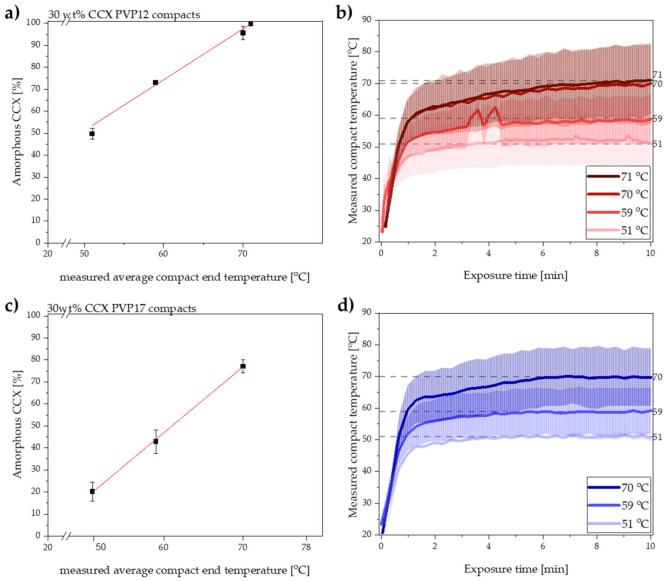
(**a**) Degree of amorphization for CCX [%] plotted as a function of the measured average compact end temperature [°C] after 10 min of microwave radiation of compacts containing PVP12. The red line indicates the linear extrapolation to obtain 100% amorphization. Mean ± SD (*n* = 3). (**b**) Measured compact temperature of the compacts containing PVP12 exposed to microwave radiation for 10 min at the measured average compact end temperature of 51, 59, 70 and 71 °C. Interpolated mean ± SD (*n* = 3). The dotted grey lines indicate the measured average compact end temperature. (**c**) Degree of amorphization for CCX [%] plotted as a function of the measured average compact end temperature [°C] after 10 min of radiation of compacts containing PVP17. The red line indicates the linear relationship. Mean ± SD (*n* = 3). (**d**) Measured compact temperature of the compacts containing PVP17 exposed to microwave radiation for 10 min at the measured average compact end temperatures of 51, 59, 70 °C. Interpolated mean ± SD (*n* = 3). The dotted grey lines indicate the measured average compact end temperature.

Compacts containing PVP12 were initially exposed to 10 min of microwave radiation resulting in measured average compact end temperatures of 51, 59 and 70 °C. Immediately after exposure to microwave radiation, the degree of amorphization was determined by transmission Raman spectroscopy. Figure 1a shows the obtained degree of amorphization for CCX plotted as a function of the measured average compact end temperature. The experimentally determined data points for the degree of amorphization of CCX, at the three different temperatures, were linearly correlated with a coefficient of determination of (R^2^) > 0.974. Based on the high R^2^ value and the assumption that the rate of amorphization can be described by the Noyes–Whitney equation (Equation (1)), the linear correlation was extrapolated to estimate the compact end temperature at which 100% amorphization of CCX would be achieved. The extrapolation of the linear correlation predicted that compacts containing PVP12 could be fully amorphized when exposed to microwave radiation for 10 min with a measured average compact end temperature of 71 °C. Hence, the set temperature of the microwave oven was increased and compacts containing PVP12 were exposed to microwave radiation for 10 min to experimentally validate the predicted data point. It was found that the exposure of compacts containing PVP12 to microwave radiation for 10 min with a measured average compact end temperature of 71 °C did indeed result in complete amorphization (99.7 ± 0.8%, *n* = 3). These results support the hypothesis of a linear correlation between temperature and rate of amorphization upon exposure to microwave radiation, at temperatures above the *T_g_* of the polymer.

**Figure 2:**
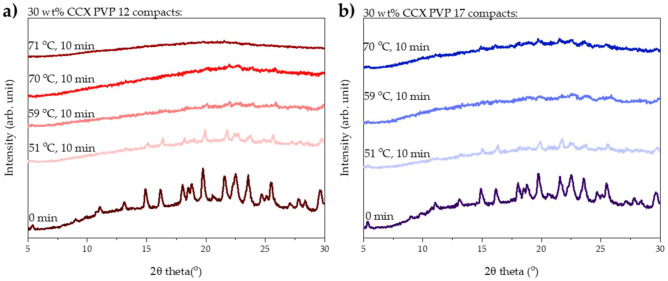
XRPD diffractograms of compacts before and after exposure to 10 min of microwave radiation. (**a**) Compacts containing 30 wt% CCX and PVP12 exposed to microwave radiation at a measured average compact end temperature of 51, 59, 70 and 71 °C. (**b**) Compacts containing 30 wt% CCX and PVP17 exposed to microwave radiation at a measured average compact end temperature of 51, 59 and 70 °C.

The degree of amorphization determined by transmission Raman spectroscopy was qualitatively confirmed by X-ray powder diffraction. From the diffractograms shown in Figure 2a, it can be seen that complete amorphization was obtained with a measured average compact end temperature of 71 °C, as the diffractogram showed a halo.

The temperature recordings obtained during exposure to microwave radiation of compacts containing PVP12 are shown in Figure 1b. It can be seen that the measured average end temperature of the compacts was reached already after 4–8 min of exposure to microwave radiation.

It is suggested that the rate of amorphization follows the trend of the temperature recording; i.e., the rate of amorphization is expected to increase relatively fast during the initial steep temperature increase and subsequently settles at constant level, depending on the reached end compact temperature (Figure 1b) [2]. With respect to changes in rate of amorphization during the exposure to microwave radiation, it is also important to mention that the viscosity of the polymer will change as a function of temperature [6], but that it is also dependent on the degree of amorphization, as more drug dissolved into the polymer will increase its viscosity [6]. Here, however, it is suggested that the change in the viscosity of each polymer, i.e., PVP12 and PVP17, during exposure to microwave radiation had a minimal effect on the rate of amorphization, as the temperatures were above the *T_g_* of the polymer (throughout the study), and the largest change in viscosity is observed at temperatures around the *T_g_* value. Furthermore, the temperatures chosen in the present study were relatively close to each other (max. 20 °C apart), which means that the viscosity difference was minimal (logarithmic dependency of the viscosity on the temperature) and therefore is expected to have had a non-significant effect on the rate of amorphization [30].

Thermal analysis was used to characterize the ASD formed upon exposure of compacts containing PVP12 to microwave radiation for 10 min at a measured average compact end temperature of 71 °C. Based on the *T_g_* measurement, it was found that the ASD was homogenous, as characterized by a single *T_g_* at 55.1 ± 1.1 °C, (*n* = 2).

### 3.3. Exposure to Microwave Radiation of Compacts Containing PVP17

Compacts containing PVP17 were exposed to microwave radiation at measured average compact end temperatures of 51, 59 and 70 °C. In contrast to the compacts containing PVP12, complete amorphization has so far not been described for compacts containing the higher molecular weight/viscosity grades of PVP, i.e., PVP17. As the *T_g_* and the viscosity of PVP17 are higher as compared to PVP12, it was suggested that a higher temperature was needed to reach complete amorphization for compacts prepared with PVP17 compared to PVP12.

After exposing the compacts containing PVP17 to microwave radiation, the degree of amorphization was directly determined using transmission Raman spectroscopy. The results for the degree of amorphization after exposure to microwave radiation for 10 min at the three different measured average compact end temperatures are shown in Figure 1c with the temperature profiles depicted in Figure 1d. As for the PVP12 compacts, the experimentally determined degree of amorphization was linearly correlated with the measured average compact end temperature for the compacts containing PVP17 (R^2^ > 0.999). Again, the linear relationship was extrapolated to estimate at which temperature 100% amorphization could be achieved.

The calculated temperature to reach the complete amorphization was at a compact temperature of 78 °C, which was above the maximum compact temperature reachable with the present experimental setup and compact composition. Hence, the predicted compact temperature value to obtain a fully amorphous ASD for compacts containing PVP17 could not be experimentally validated with the current experimental setup.

As for the compacts prepared with PVP12, the degree of amorphization was qualitatively confirmed using XRPD. From Figure 2b, it can be seen that no fully amorphous solid dispersion was obtained from compacts containing PVP17.

For the PVP17 compacts, the compact end temperature was reached within 3–6 min of microwave radiation. Comparing the temperature profiles obtained for compacts containing PVP17 (Figure 1d) with compacts containing PVP12 (Figure 1b), it is suggested that differences in the initial average heating rates (t~0–1 min) can be seen. Despite the similar, or even faster, initial heating rate at a measured average compact end temperature of 70 °C, the rate of amorphization for compacts containing PVP17 was slower than for compacts containing PVP12 at similar temperatures (compare Figure 1a,c). As water acted not only as the dielectric heating source inside the compacts upon exposure to microwave radiation, but also as a plasticizer, it is suggested that the different degree of interaction of the water with the polymer played a crucial role here. As suggested above, loosely bound water, which is more mobile compared to tightly bound water, will lead to a faster heating rate; however, it also leads to a faster dehydration process, which in turn will increase the *T_g_* of PVP17 and the forming ASD faster as compared to PVP12 and the forming ASD. It is suggested that this increase in the *T_g_* of the polymer resulted in a slower amorphization for compacts containing PVP17 compared to PVP12.

Following the same procedure as for the PVP12 compacts, thermal analysis of compacts containing PVP17 exposed to microwave radiation at 70 °C for 10 min (highest measured average compact end temperature) was performed. It was revealed that a non-homogenous two-phase system was obtained, with two *T*_g_s, i.e., one for a drug-rich phase and one for a polymer-rich phase. The *T_g_* for the drug-rich phase and polymer-rich phase was 60.1 ± 1.1 °C (*n* = 2) and 97.4 ± 15.2 °C (*n* = 2), respectively. The detection of a *T_g_* above 70 °C for the polymer-rich phase indicates that complete amorphization was not possible with the present settings, as this compact temperature would not be reached.

### 3.4. Influence of Water Evaporation on the Compact Temperature during Exposure to Microwave Radiation, and the T_g_ of the Polymer

Even though a fast heating rate is beneficial for *in situ* amorphization, as the rate of amorphization increases with increasing temperature (Equation (1)), a fast heating rate will also cause the compact water to evaporate faster. As a result of water evaporation, the *T_g_* of the polymer and forming ASD will increase (Table 1 and Section 3.3), the mobility of the polymer will decrease and eventually the dielectric heating source will be lost, which is suggested to ultimately limit the *in situ* amorphization process.

As described in Section 3.1, it is suggested that the two polymers, PVP12 and PVP17, interacted differently with sorbed water, i.e., the water was bound to a different degree, which would lead to an initial higher heating rate for compacts containing PVP17, as the water was less tightly bound in these compacts as compared to compacts containing PVP12. When comparing the initial heating rates (t~0–1 min) observed for compacts containing PVP12 and PVP17, at the measured average compact end temperature of 70 °C (Figure 1b,d), a higher initial average heating rate for compacts containing PVP17 was observed (though determined to not be statistically different, *p* > 0.05).

Following the initial steep compact temperature increase, the heating rate decreased for both compact compositions, and the compact temperature approached a plateau. It was found that after the initial heating, the heating rate slowed down to 1.1 °C/min for compacts containing PVP17 and to 1.2 °C/min for compacts containing PVP12 (t~1–10 min). This shift in the observed heating rates is suggested to be caused by water evaporation, indirectly indicating that water evaporated faster from the PVP17 compacts. These results correlate well with the suggested degree of interaction of water with PVP12 and PVP17, as PVP12 showed a higher degree of tightly bound water compared to PVP17 (see Section 3.1).

**Figure 3:**
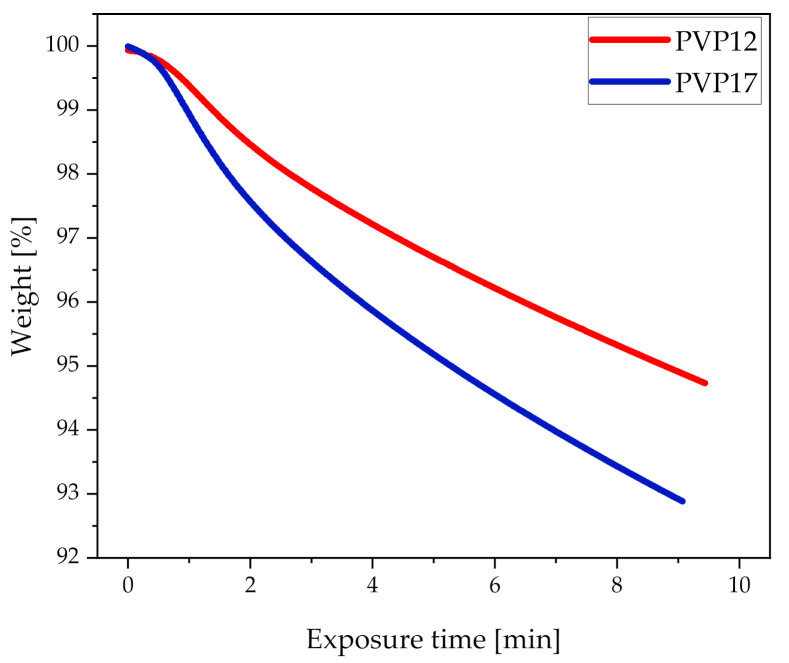
Pure conditioned polymers, PVP12 (red) and PVP17 (blue) exposed to TGA heating with the heating rates obtained during exposure of compacts containing 30 wt% CCX and PVP12 or PVP17 to microwave radiation for 10 min at a measured average compact end temperature of 70 °C (*n* = 1).

In order to determine how the different heating rates affected the water evaporation from compacts containing PVP12 and PVP17, at the highest common measured average compact end temperature (70 °C), the pure conditioned polymers were exposed to the same heating rates obtained during exposure to microwave radiation on the TGA (Figure 3). It can be seen in Figure 3 that PVP17 did indeed lose the water faster, as compared to PVP12. Hence, it is suggested that complete amorphization could not be achieved for compacts containing PVP17 using sorbed water as a dielectric heating source, as the sorbed water evaporated too fast. Due to a low degree of interactions between the sorbed water and PVP17, the sorbed water evaporated quickly during exposure to microwave radiation, which resulted in a partial loss of the plasticizer, which increased the *T_g_* of the polymer and forming ASD to temperatures which could not be reached, as also the dielectric heating source was partly lost. Based on these results, it is suggested that, when using water as a dielectric heating source for microwave-induced *in situ* amorphization, some polymers are less suitable than others. Still, it is important to remember that this study only presents a case study based on one model drug and two different grades of PVP, PVP12 and PVP17, i.e., further studies are needed to investigate if the observed behavior also applies for other polymers, including higher molecular weight polymers.

## 4. Conclusions

This study showed that during microwave-induced *in situ* drug amorphization of compacts containing 30 wt% CCX and PVP12 or PVP17, the rate of amorphization was linearly dependent on the compact end temperature in the range 51–70 °C. For PVP12 compacts, a fully amorphous ASD was obtained following exposure to microwave radiation for 10 min at a measured average compact end temperature of 71 °C. Compacts containing PVP17 could not be fully amorphized upon exposure to microwave radiation with the present experimental setup and compact composition. The limitation for *in situ* amorphization for compacts containing PVP17 was found to be the (sorbed) water evaporating so fast that complete amorphization was not achieved before the *T_g_* of the polymer and forming ASD had increased above the measured average compact end temperature. Hence, using sorbed water as a dielectric heating source, it is recommended to use a polymer with an initial low *T_g_* and a low molecular weight, as well as a high degree of tightly bound water to achieve 100% amorphization upon exposure to microwave radiation.

## Figures and Tables

**Table 1:** Glass transition temperatures of the polymers PVP12 and PVP17; mean ± SD (*n* = 2).

Polymer	*T_g_* (Water-Free)	Bulk *T_g_* (Including Water in the Bulk PVP)	*T_g_* after Conditioning	Theoretical Bulk *T_g_* (Including Water in the Bulk PVP)	Theoretical *T_g_* after Conditioning
PVP12	105.3 ± 0.5 °C	39.7 ± 0.2 °C	−11.6 ± 0.9 °C	75.1 °C	−2.5 °C
PVP17	120.6 ± 0.1 °C	54.5 ± 2.9 °C	3.2 ± 0.6 °C	64.5 °C	1.2 °C

## Data Availability

The data are available upon request from the authors.
